# Influence of Experimental Conditions on Electronic Tongue Results—Case of Valsartan Minitablets Dissolution

**DOI:** 10.3390/s16091353

**Published:** 2016-08-23

**Authors:** Małgorzata Wesoły, Anna Kluk, Małgorzata Sznitowska, Patrycja Ciosek, Wojciech Wróblewski

**Affiliations:** 1 Department of Microbioanalytics, Warsaw University of Technology, Noakowskiego 3, Warsaw 00-664, Poland; wuwu@ch.pw.edu.pl; 2 Department of Applied Pharmacy, Gdańsk Medical University, Al. Gen. J. Hallera 107, Gdansk 80-416, Poland; ankl@gumed.edu.pl (A.K.); msznito@gumed.edu.pl (M.S.)

**Keywords:** electronic tongue, potentiometric sensors, drug release, dissolution test, minitablets, valsartan, taste-masking

## Abstract

A potentiometric electronic tongue was applied to study the release of valsartan from pharmaceutical formulations, i.e., minitablets uncoated and coated with Eudragit E. Special attention was paid to evaluate the influence of medium temperature and composition, as well as to compare the performances of the sensor arrays working in various hydrodynamic conditions. The drug dissolution profiles registered with the ion-sensitive electrodes were compared with standard dissolution tests performed with USP Apparatus 2 (paddle). Moreover, the signal changes of all sensors were processed by principal component analysis to visualize the release modifications, related to the presence of the coating agent. Finally, the importance and influence of the experimental conditions on the results obtained using potentiometric sensor arrays were discussed.

## 1. Introduction

The majority of medicines are designed for oral administration, which is the most acceptable to patient and the most frequently chosen route of administration due to its ease of production and application. Moreover, it is one of the most physiological routes of entry of the substance to the human body [1,2]. The rate at which active pharmaceutical ingredients (APIs) will be absorbed to the bloodstream depends on the releasing of the APIs from the pharmaceutical product, the dissolution or solubilisation under physiological conditions, and the permeability across the gastrointestinal tract [3].

Among various studies of solid oral dosage forms, the dissolution test is one of the most important. It demonstrates the release rate of the active substance from the solid matrix or from the particles after its disintegration. The amount of the substance dissolved in the medium simulating physiological fluids is determined. The test is used in all phases of the formulation development, in the monitoring of the production process and as a quality control tool, to detect the physicochemical changes in the pharmaceutical formulations. It can also be used for the prediction of the in vivo performance of pharmaceutical products [4]. The pharmacopoeial apparatus are recommended for standard dissolution tests of solid dosage forms; among them paddle or basket apparatus are the most common. A tablet or a capsule is introduced to the stirred dissolution medium and at certain time points the medium samples are collected from the vessel and the quantity of API is determined usually by high-performance liquid chromatography or UV-VIS techniques [5]. Typical dissolution tests are generally performed at the temperature of 37 °C and under “sink conditions”, that is, at least 3–10 times the saturation volume, to ensure the complete drug release from the dosage form. Further experimental testing conditions are standardized: the pH of dissolution medium is set between pH 1–8, stirring speed must not exceed 150 rpm, and the volume of the dissolution medium is at least 500 mL, typically 900 mL [6]. However, these parameters differ from the physiological conditions.

The appearance of novel drug delivery systems and growing demand for fast, reliable analysis of pharmaceutical products lead to the development of analytical methods and tools for the characterization of their properties. One such promising analytical device is the electronic tongue (ET). Electronic tongues are devices composed of sensor arrays and pattern recognition systems that are able to distinguish complex liquid samples and to recognize their characteristic properties [7]. The commercially available electronic taste sensing systems are supplied by Japanese Insent (Taste Sensing System) and French AlphaMOS (αASTREE); also, various laboratory prototypes have been developed. Their applications to the analysis of food samples, environmental monitoring, and bioprocess control have been widely described in numerous reviews [8,9,10]. Moreover, the use of ETs for pharmaceutical purposes has grown within the recent years [11].

The taste of pharmaceutical products is one of the major factors affecting patient adherence, especially in paediatric formulation. Hence, one of the major applications of the electronic tongue is the assessment of taste-masking efficiency. This bitter taste-masking is realized by various techniques, such as the addition of sugars or sweeteners [12,13], microencapsulation [14], hot melt extrusion [15], or complexation of API [16]. In contrast to the adult population, children do not have access to tablets which can be easily coated with taste-masking polymers. The reason is poor ability of children to swallow tablets. Only recently has the technology of small tablets, called minitablets (1–3 mm diameter), been developed and it was demonstrated that such small tablets can be swallowed by children at the age 0.5–3 years, when administered with a drink or with food [17,18].

Various comparisons of taste-masking techniques, measurement protocols, analytical procedures, as well as comparisons between commercially available systems applied to pharmaceutical analysis were reported [19,20]. Novel drug delivery systems, such as orally disintegrating tablets, film formulations, and microparticles, were evaluated using electronic tongues [21,22,23]. Additionally, independent interlaboratory tests of the performance of the Taste Sensing System TS5000Z (Insent, Intelligent Sensor Technology, Inc., Kanagawa, Japan) [21] or six different ET systems (two commercially available systems and four laboratory prototypes) were recently presented [24]. The correlation between the results provided by ETs and the assessment of the taste of pharmaceutical products performed by a human test panel, required obtaining reliable data, and was reported in numerous studies [22,23,25]. Finally, the bitterness detection was also provided by an in vivo bioelectronic tongue in combination with brain-machine interface technology [26].

Regarding the fact, that dissolution of API and the disintegration of dosage forms are significantly influenced by the temperature, pH, medium composition, ionic strength, and hydrodynamics, the experimental conditions of measurements performed by ET should be standardized as for reference methods. Some qualification protocols for taste evaluation using ET systems were established, but only for commercial systems. Usually, purified water was depicted as the dissolution medium [27] while, in a few papers, the temperature of the dissolution medium (water) was set to 37 °C and the stirring speed was 25 rpm [28] or 100 rpm [29]. The impact of the sample temperature on the results obtained by αASTREE (i.e., temperature correction of ET measurements) was considered only in one report for model solutions and foodstuff samples analysis [30]. Moreover, specificity, linearity, range, accuracy, precision, detection, and quantification limits, as well as robustness, were determined for αASTREE and Taste Sensing System SA402B; additionally, the measurement protocols were provided by the manufacturers [31,32]. Nevertheless, no standardization protocols of measurements and experimental conditions for laboratory prototypes exist. Recently, our group presented the first comparison of the performances of ETs (equipped with three different sensor arrays) working in various hydrodynamic conditions during the analysis of pharmaceutical samples [33].

In conclusion, despite the fact that the measurement conditions strongly influence the obtained results of dissolution tests using ETs, such an issue was not studied and discussed so far. Therefore, the influence of medium composition (pH, ionic strength), medium temperature, and hydrodynamic effects on ET results of dissolution of pharmaceutical samples is evaluated in this work. For comparison a standard pharmacopoeial dissolution was performed. Minitablets containing valsartan (uncoated cores and units coated with Eudragit E) are used as model pharmaceutical samples studied by ET systems. Valsartan is an angiotensin II receptor antagonist regulating the cardiovascular functions, used in hypertension management [34,35,36]. Eudragit E is a film forming agent from the polymethacrylates family, soluble in acidic pH of the stomach but insoluble at pH > 5, i.e., in saliva, making it suitable for taste-masking.

## 2. Experimental

### 2.1. Chemicals and Membrane Materials

All used inorganic salts were of analytical grade, high-molecular-weight poly (vinyl chloride) (PVC), plasticizers, lipophilic salts, and ionophores were obtained from Fluka (Selectophore). All solutions were prepared in redistilled water. The membranes contained 1%–3% *w*/*w* electroactive components, 66% *w*/*w* plasticizer, and 31%–33% *w*/*w* high-molecular-weight PVC (Table 1).

The membranes components (200 mg in total) were dissolved in 2 mL of freshly distilled tetrahydrofuran. The method of the membranes preparation and the electrodes conditioning were the same as previously reported [14]. The constructed ISEs were preconditioned at least 24 h. All measurements were carried out in cells of the following type: Ag, AgCl; KCl 3 M|CH_3_COOLi 1 M |sample solution‖membrane‖internal filling solution; AgCl, Ag. Potentiometric multiplexer (EMF 16 Interface, Lawson Labs Inc., Malvern, PA, USA) was used for electromotive force (EMF) measurements.

### 2.2. Sensor Arrays and Electronic Tongue Measurements

Two electronic tongue systems with similar sensor arrays were applied in order to evaluate ET performance in various hydrodynamic conditions. Sensor array of the ET for batch measurements was composed of 16 classical ion-selective electrodes (ISEs) of various cross-sensitivity (according to different electroactive additives), whereas sensor array of the flow-through ET consisted of 10 miniaturized ISEs (two specimens for each type, see Table 1). The design of the modular flow system is a subject of a Polish patent application and was previously reported [33]. The sample was introduced into the flow-through sensor array (flow-rate 1 mL/min) by a peristaltic pump (Minipuls 2, Gilson). The solution was forced to pass 0.2 μm filter before reaching the sensor array and was recirculated to maintain a constant volume.

The calibration curves of the constructed electrodes towards valsartan and Eudragit E were examined in batch conditions. Regarding the poor solubility of valsartan in water, the calibration curves were recorded by measuring the EMFs in the concentration range 10^−5^–10^−3.5^ M.

**Figure 1:**
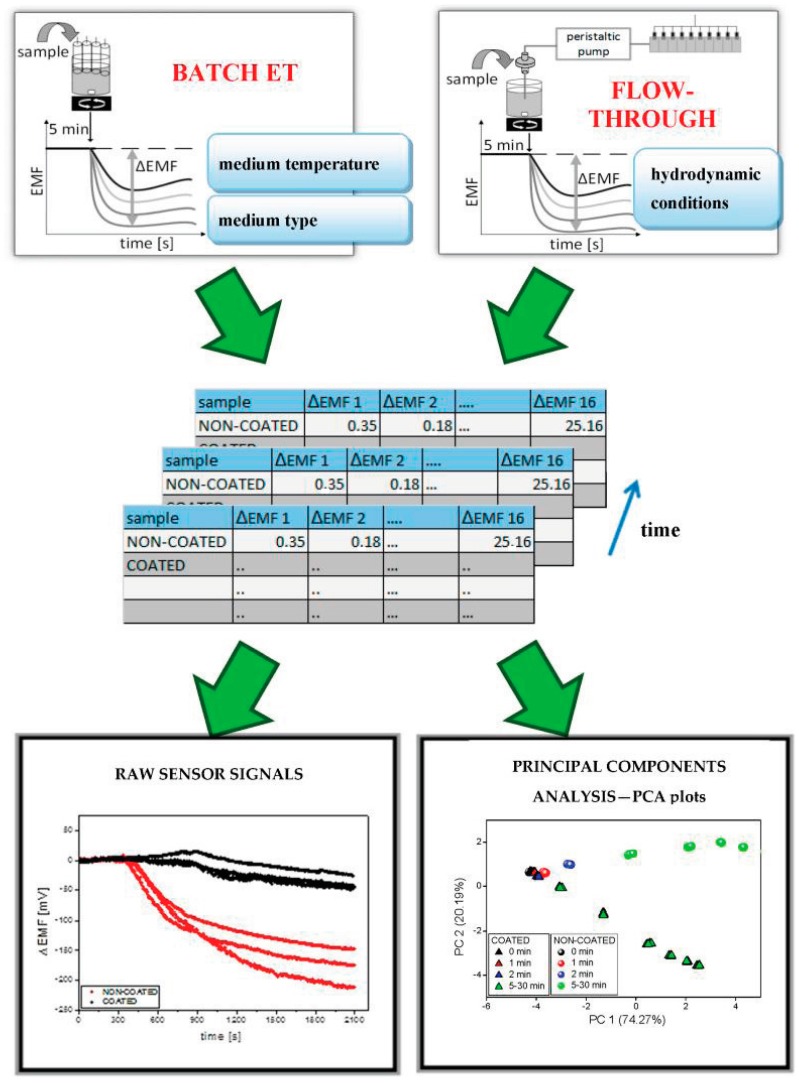
Acquisition and analysis of ET signals.

Measurement procedure was developed for dissolution tests of pharmaceutical formulations. After the stabilization of potentiometric signals of ISEs immersed in pure medium (100 mL) for 5 min, 10 minitablets were added into the medium and the release of API as well as excipients from the formulations was observed in terms of the changes of sensor signals (ΔEMF) in time. The EMF changes were also subjected to multivariate analysis to visualize the release modifications by coating (Figure 1). Nine time points of signal measurement were chosen within a time range 0–30 min (after the introduction of the minitablets into dissolution medium) and the results were processed by principal component analysis (PCA).

The experiments were carried out in triplicate under various conditions:

- batch measurements in two temperature values: room temperature (20–25 °C) or 37 °C;
- batch measurements in three medium types: deionized water, phosphate buffer pH 6.9 (prepared according to Ph.Eur., diluted to 1 mM), and artificial saliva pH 6.8 (0.17 M NaCl, 1.4 mM KH_2_PO_4_, 6.6 mM NaH_2_PO_4_);
- two systems working in various hydrodynamic conditions: batch ET and flow–through ET.

### 2.3. Pharmaceutical Formulations and Reference Dissolution Studies

Two minitablets formulations containing valsartan, i.e., uncoated and coated with Eudragit E were tested. Biconvex minitablets (diameter 2.5 mm, weight 12.5 mg), containing 5 mg of valsartan (40% *w*/*w*) were produced. The following excipients were other components of minitablets: lactose (27.5%), microcrystalline cellulose (27.5%), povidone K30 (2.5%), croscarmellose sodium (2%), and magnesium stearate (0.5%). For coating, 8% *w*/*w* aqueous dispersion of Eudragit E PO was used in a fluid-bed system (InnoJet AirCoater, Steinen, Germany) to achieve coating with 100 μm of thickness. Apart from polymer the coating mixture contained: dodecylsulfate sodium (0.9%), stearic acid (13%), talc (43%), and colorant-azorubine (0.003%).

Dissolution tests were performed using a paddle apparatus (Erweka DT-820, with autosampler, Heusenstamm, Germany) according to the Ph.Eur. monograph. Sixteen minitablets (either coated or uncoated) were used as a single dose and were submerged in 900 mL of phosphate buffer (pH 6.5, 0.05 M). The temperature of the dissolution medium was maintained either at 25 ± 0.5 °C or 37 ± 0.5 °C. The samples were collected in particular time points (10, 20, 30, 45, 60, 120 min) and the amount of dissolved drug was determined by HPLC method with UV-VIS detection, at a wavelength of 215 nm. The mean values were recalculated as the percent of valsartan dose released and presented graphically. Dissolution tests were performed in triplicate for each formulation and process condition.

## 3. Results and Discussion

### 3.1. Reference Dissolution Studies

Nowadays, minitablets have a great potential as a novel solid drug delivery system. Due to its small size, they can facilitate swallowing especially in children [37]. Eudragit E is a cationic copolymer based on dimethylaminoethyl methacrylate, butyl methacrylate, and methyl methacrylate, used as a coating agent due to the ability of pH-dependent solubility. It is soluble in a gastric fluid (below pH 5) and used as a popular taste-masking agent in pharmaceutical formulations.

**Figure 2:**
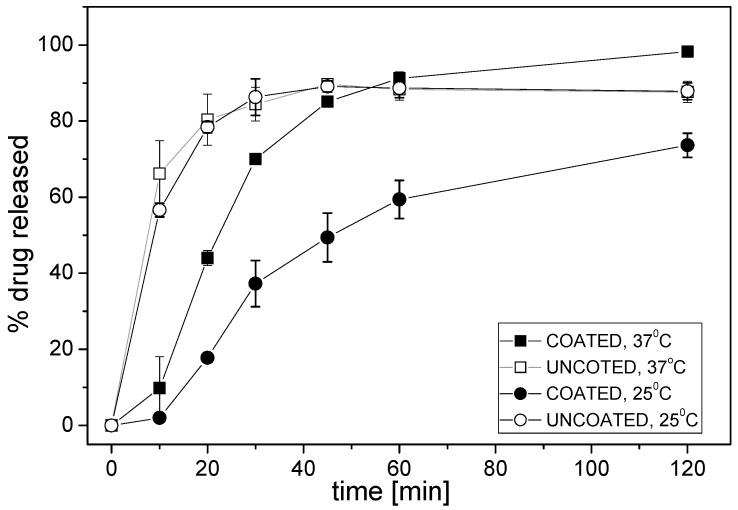
Dissolution profiles of valsartan from minitablets coated with Eudragit E or without coating (phosphate buffer pH 6.5; mean ± SD; *n* = 3).

Valsartan minitablets—uncoated and coated with Eudragit E were compared. First, classical dissolution testing was performed according to the pharmacopoeial (Ph.Eur./USP) methodology, using paddle apparatus. Dissolution profiles for valsartan from both types of minitablets are presented in Figure 2.

Different release rate of valsartan from various minitablets was observed in phosphate buffer pH 6.5 during the first 45 min—the release of API from minitablets coated with Eudragit E was slower than from uncoated minitablets. The largest difference was observed during the first 10 min: at 37 °C the formulation with Eudragit E released 10% of API, whereas more than 65% of the drug was released from the other formulation. The kinetics of drug release from the coated minitablets was determined by the temperature of the medium, i.e., the degree of the release of API was approximately 1.5–2-fold larger at 37 °C, while the effect of the temperature was negligible for the uncoated minitablets. The obtained results arise from different rates of disintegration of the polymer coating at various temperatures.

### 3.2. Performances of the Potentiometric Sensor Arrays

The sensor arrays comprise PVC membranes electrodes based on an appropriate lipophilic salt, exhibiting generic anion (AS, PS) or cation (CS) response (Table 1). Moreover, carbonate-selective electrodes (CARB) were included as additional anion-sensitive sensors exhibiting a selectivity pattern [38], which differs from that observed for AS and PS electrodes. Before electronic tongue measurements, the calibration curves of the electrodes towards both valsartan and Eudragit E were evaluated. It is a crucial step before every experiment with electronic tongue system, verifying the applicability of the sensors to the performed analysis.

**Figure 3:**
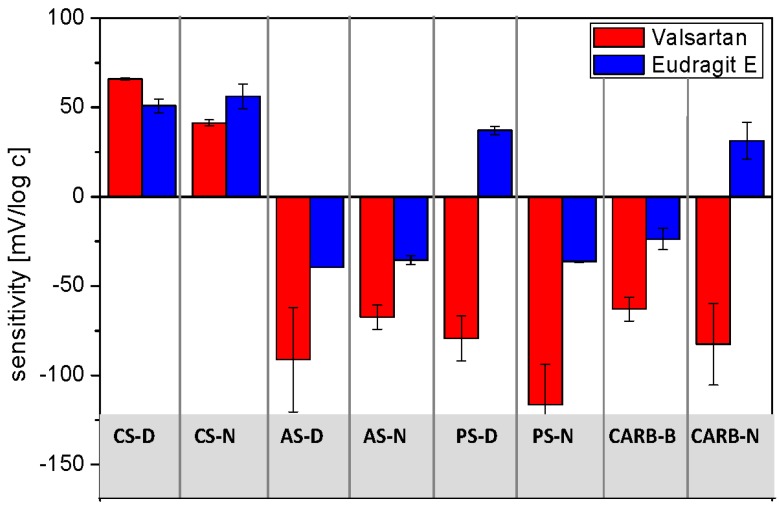
Sensitivity of sensors towards Valsartan and Eudragit E (mean ± SD; *n* = 3).

According to our expectations the measured potentiometric signals originated from non-selective interactions and depending on the electroactive component, cationic, or anionic sensitivity of the examined ISEs were observed (Figure 3).

Since valsartan is a weak acid (pK_a_ = 3.9 and 4.7), the anion-sensitive electrodes were found to respond to this API; in general the linear range of calibration curves exhibited slope exceeding Nernstian values, which can be explained by a more complex response mechanism. Probably, since the electrodes were conditioned in solution of discriminated ions (diluted NaCl solution), the membranes composition changed in time with increasing concentration of target molecules in the sample due to the intramembrane ion fluxes. This process influences the electrode potential during the experiment and may lead to non-Nernstian behavior [39]. On the other hand, cationic responses with lower sensitivities were recorded for the electrodes based on KTFPB. In the case of Eudragit E, cationic responses of the CS-D and CS-N electrodes towards this cationic copolymer, as well as weak anionic responses of the anion-sensitive electrodes were observed. Only the cationic responses of electrodes PS-D and CARB-N were unexpected and it is difficult to elucidate the origin of this result. Finally, it is worth noting that the performances of the constructed electrodes were repeatable during at least two months.

### 3.3. Electronic Tongue Dissolution Studies under Various Conditions

Since the constructed electrodes exhibited different sensitivity patterns towards valsartan and Eudragit E, the sensor arrays were applied for the dissolution studies of minitablets containing valsartan and to evaluate the impact of various experimental conditions on the dissolution profiles obtained with the use of ET system.

**Figure 4:**
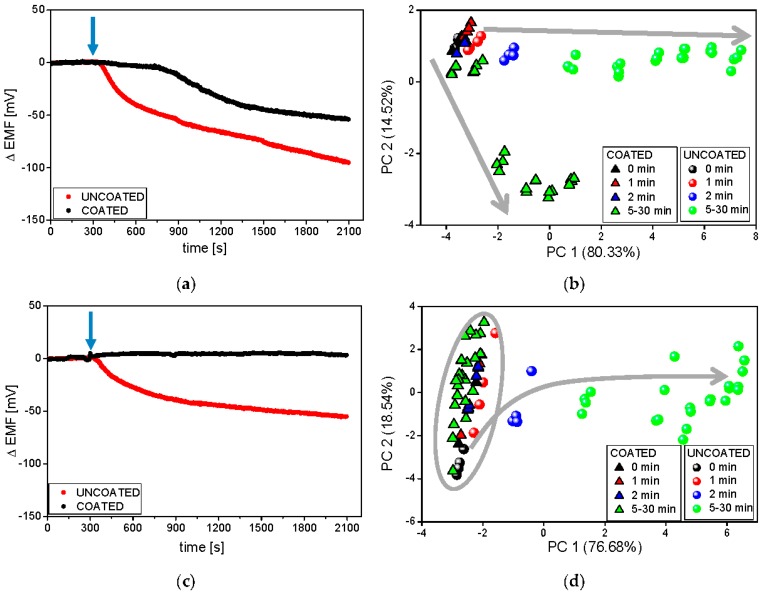
Raw signals of the PS-N electrode and respective PCA plots during the release process from minitablets uncoated and coated with Eudragit E, carried out in phosphate buffer pH 6.9 at 37 °C (**a**,**b**) and room temperature (**c**,**d**). The blue arrows represent the moment of the introduction of minitablets into the dissolution medium. Grey arrows represent following time points.

The temperature of the dissolution medium was the first investigated experimental factor possibly influencing the results. Typical raw sensor signals recorded during the release experiments at two temperatures of the dissolution medium (room temperature and 37 °C) are presented on the example of the PS-N electrode (Figure 4a,c).

It must be underlined that temperature influences the obtained results due to the temperature dependence of the electrode potential according to the Nernst equation, drug release kinetics, disintegration time of the studied pharmaceutical formulations, and solubility of API, coating polymer, and other excipients. Therefore, it is evident that depending on the temperature, different dissolution profiles were obtained for coated and uncoated minitablets. A strong drop of the sensor signal in time caused by the release of valsartan was observed for uncoated formulation in both temperatures; in contrast, the electrode potential remained unchanged for coated minitablets in the lower temperature. These results correlate well with the reference dissolution studies (see Figure 2), where the high slowdown of minitablets disintegration was noticed at 25 °C. Moreover, the faster API release from both formulations was observed at higher temperature. The largest difference was noticed during the first 10 min, i.e., uncoated formulation started immediately to release API, whereas the release from the coated minitablets was visible with a lag time of approximately 10 min, which is consistent with the presented above reference studies. Concluding, the dissolution profiles obtained using potentiometric detection confirmed that the temperature alters the kinetics of the drug release (its rate and degree). Similar conclusions can be drawn on the basis of dissolution profiles recorded for the other electrodes of the tested sensor array.

In the next step, the pattern of sensor responses was analyzed with the use of PCA method in order to better visualize the temperature dependence of the rate of drug release. Strong influence of the temperature on the chemical images on 2D-PCA plot can be seen comparing Figure 4b,d. Generally, the releasing process run can be observed along the first principal component (PC1), which captured the most amount of variance in whole data set. For both temperatures, significant differences between the release of valsartan from coated and uncoated minitablets, as well as the largest change of chemical image for uncoated minitablets were noticed. Separable and distinct clusters were visible at higher temperature, which was related with better distinguishing between samples representing various time points due to higher changes of sensor signals in time. Moreover, the chemical image of the coated formulation 30 min after the addition of minitablets is close to the chemical image of uncoated formulation at 5/10 min time point, when only PC1 is considered. It correlates well with the reference dissolution studies, where ca. 70% of API was released after 30 min and 10 min for coated and uncoated minitablets, respectively. On the other hand, almost no difference in chemical images related to the release of API from coated minitablets was noticed at lower temperature (the chemical images for all time points are placed very close in the PCA space, forming the same cluster with the chemical images of uncoated minitablets for 0/1 min time points). The presented results showed, that the measurements performed at higher temperature are more advantageous, providing more distinguishable chemical images and higher accordance with the reference dissolution results.

Deeper understanding of influence of temperature effects on ET results was performed by the correlation of amount of API released and distance in PC1-PC2 space calculated from “0 min” time point (Euclidean distance between centroids of clusters representing respective time points). High correlation was found for the results obtained by ET at room temperature and classical dissolution test in 25 °C (R^2^ = 0.884; *p* = 0.0026) as well as for ET in 37 °C and classical dissolution test in 37 °C (R^2^ = 0.889; *p* = 0.0024). However, when comparing ET results for lower temperature and dissolution test results obtained in 37 °C, which is common in most of the works dealing with ET analysis of pharmaceuticals, correlation was much lower and less statistically significant (R^2^ = 0.540; *p* = 0.048). Thus, it is noticeable that ET can correctly trace the amount of the released API, but comparison shall be performed in the same temperature conditions.

**Figure 5:**
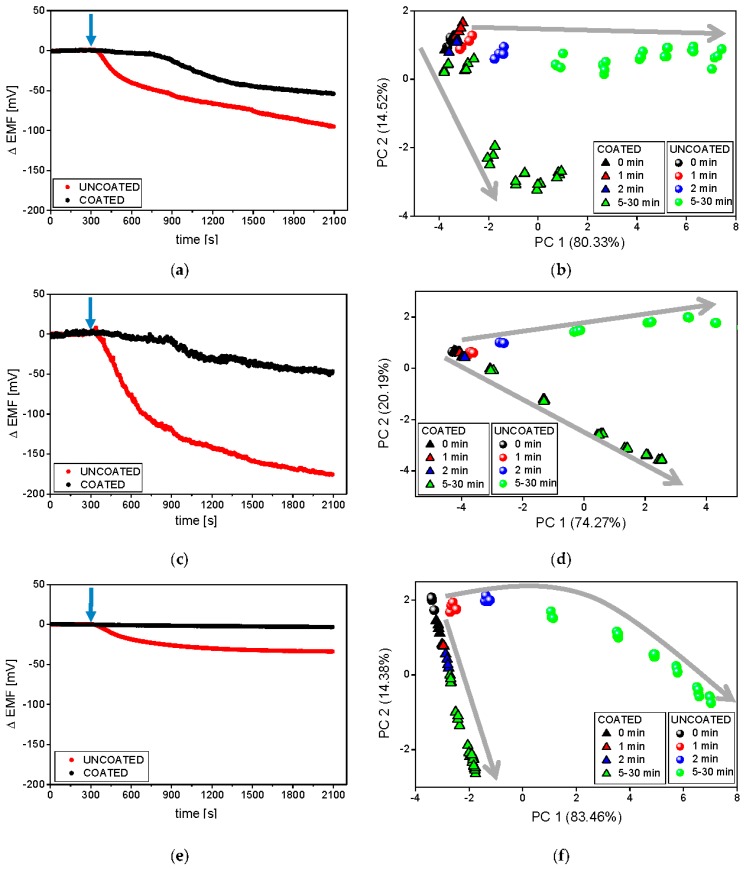
Raw signals of PS-N electrodes and respective PCA plots during the release process carried out at 37 °C in phosphate buffer at pH 6.9 (**a**,**b**), deionised water (**c**,**d**), and artificial saliva at pH 6.8 (**e**,**f**). The blue arrows represent the moment of the introduction of minitablets into the dissolution medium. Grey arrows represent following time points.

Secondly, the influence of the composition of dissolution medium on the results provided by ET was evaluated. The experiments were carried out at 37 °C in three different dissolution media: purified water, phosphate buffer at pH 6.9, and artificial saliva pH 6.8. In general, purified water was applied as the medium during ET analysis of pharmaceuticals, whereas the reference studies were performed in buffers (dissolution studies) or saliva (in vitro studies). This fact can hinder the interpretation of the results, since ionic strength and pH strongly influence the process of disintegration/dissolution, as well as alter the signal of potentiometric sensors. Therefore, it should be emphasized that different dissolution profiles may be recorded changing these parameters, i.e., pH and ionic strength of dissolution media. Raw sensor outputs of exemplary PS-N electrodes in various dissolution media are presented in Figure 5a,c,e.

In all cases, clear differences between the sensor signals measured during the dissolution of coated and uncoated tablets were visible. Comparable, delayed release of valsartan from the coated formulation was observed on the raw sensor signals representing experiments carried out in water and buffer solution (on the contrary, the decrease of the sensor potential was not registered during the measurements in artificial saliva). In the case of the uncoated formulation, the magnitude of the signal drop was mainly limited by the ionic strength of the solution (affecting the limit of detection of the sensors). Therefore, the highest ΔEMF changes were measured in water and the smallest in artificial saliva (accordingly, the highest signal stability was noticed in the latter case).

Thereafter, the signals of all sensors in the array were processed by PCA and the resulting chemical images of the studied samples were collected on respective 2D PCA plots (Figure 5b,d,f). According to the presented previously dissolution curves, different releasing profiles of coated and uncoated minitablets were obtained in all media. Similar run of releasing was observable along PC1; however, the end-points corresponding to coated formulations exhibited smaller changes of PC1 in comparison with those calculated for the uncoated tablets. Higher changes in PC2 were received in the case of coated minitablets (probably, PC2 reflects the increasing concentration of Eudragit in solution). A direct correlation with the reference release studies was remarked only in the case of the experiments conducted in purified water—30 min after the minitablets were placed in the medium, the chemical images of the coated formulation corresponded to chemical images of the uncoated formulation at 15 min time points (~70% of the released API in both cases, compared with Figure 2). On the other hand, the most repeatable, and the smallest signal change, related to the increasing amount of the released valsartan as well as the most compact clusters were obtained in artificial saliva (Figure 5e,f). The chemical images of the coated formulation were very close to each other at various time points and their change was observed along PC2, whereas the changes of the chemical images of the uncoated minitablets were clearly noticeable mainly along PC1 axis. Moreover, only in the case of artificial saliva, for each time point clear distinction of minitablets with and without coating can be performed. Therefore, the application of artificial saliva, i.e., medium providing high ionic background and pH 6.8 (close to the physiological conditions) seems to be the most appropriate for the discrimination of such samples, which is extremely useful from the point of view of evaluation of the taste-masking efficiency.

**Figure 6:**
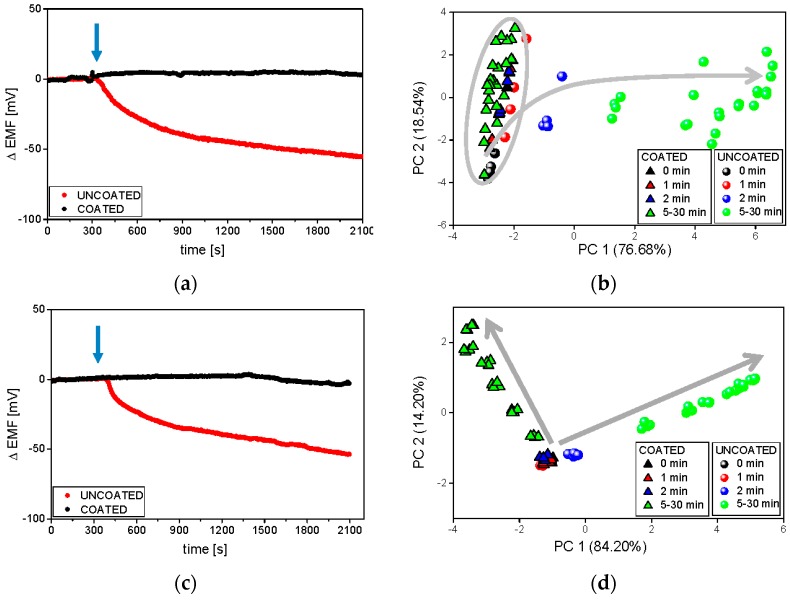
Raw signals of the PS-N electrode and respective PCA plots during the release process carried out at room temperature in phosphate buffer at pH 6.9, in various hydrodynamic conditions: batch ET (**a**,**b**), and flow-through ET (**c**,**d**). The blue arrows represent the moment of the introduction of minitablets into the dissolution medium. Grey arrows represent following time points.

At the end, the functionality of the sensor arrays working in various hydrodynamic conditions for drug release studies was compared. The measurements were carried out in room temperature in phosphate buffer using two ET systems described in the experimental section. It must be underlined that in the flow-through system the medium was forced to pass 0.2 μm filter before reaching the sensor array. As previously, raw sensor outputs of exemplary PS-N included in both systems are presented in Figure 6.

The most pronounced was the improvement of the electrode performance in flow-through conditions—the sensor readings were much more repeatable and clustering of samples became more compact. Significant changes in the sensor signal related to the release of API from uncoated formulation were immediately observed, while the releasing of valsartan from the coated minitablets was detected (after almost 20 min) only by the flow-through system. For this reason, the chemical images of all samples were easily separable after 2 min on PCA plot presenting flow measurements, on the contrary to chemical images of coated minitablets in batch conditions. The dissolution process is visible along the PC1 axis during batch experiments (uncoated samples), whereas in flow-through setup the direction of the chemical images changes can be traced along PC1 and PC2. The opposite direction of these changes for both formulations, probably due to the higher impact of Eudragit E on the signals of flow-through sensors, contributes to better distinction of samples, which is crucial for taste-masking studies. The good repeatability of the sensors signals and clear discrimination of the chemical images on PCA plot, revealed using flow-through set-up, may result from the sample filtering before measurements (which prevented the fouling of sensor membranes by any undissolved particles of minitablets). Finally, it should be stressed that the experiments were possible and made only at ambient temperature, but the development of a flow-through system enabling the measurements at 37 °C would be valuable considering the correlation with the reference studies.

## 4. Conclusions

Electronic tongue systems equipped with cross-sensitive electrodes were used to investigate the release kinetics of valsartan from minitablets coated with Eudragit E and minitablets without coating. The dissolution tests were carried out under various experimental conditions and compared with reference dissolution studies made according to the pharmacopoeial methodology. The signals of the sensor arrays were processed by PCA to better visualize the influence of the experimental conditions on the drug release results.

The results obtained using potentiometric detection confirmed that the temperature alters the rate/degree of drug release. The measurements performed at higher temperature are more advantageous: the releasing of API is traceable, chemical images are more distinguishable, and higher accordance with reference dissolution results is achieved. Therefore, taking into account that the dissolution studies in Ph.Eur./USP apparatus are made at 37 °C, the experiments with electronic tongues should also be performed at these conditions. In the majority of the articles reporting the application of ETs to the pharmaceutical analysis this issue is overlooked, while it could improve the reliability and interpretability of the results and at the same time could strengthen the correlation with standardized reference studies.

The results also indicated that, depending on the composition of the medium used during the experiments, various dissolution profiles were registered. The application of artificial saliva medium of high ionic background was the most appropriate from the point of view of evaluation of taste-masking efficiency (better distinction of samples with and without taste-masking coating), while phosphate buffer should be a chosen medium for dissolution studies (higher correlation of ET results with reference methods). Finally, the application of the flow-through detection mode improved the performances of the sensor array i.e., higher repeatability of sensor readings and better clustering of samples was achieved and could be beneficial in terms of simplification, as well as automatization of analytical procedures.

Concluding, the question of various experimental conditions and their influence on ET results was neglected during the pharmaceutical analysis done by pharmacists using commercial ET devices. However, the results obtained proved that the conditions of studies performed by an electronic tongue are crucial and should be standardized as in reference methods, where strict testing protocols and specification limits are provided by the pharmacopoeias.

## Figures and Tables

**Table 1:** Sensor arrays of the electronic tongue systems.

Electrode Type	Electroactive Component		Plasticizer	Batch ET	Flow-Through ET
CS-D	KTFPB		DOS	+	−
CS-N	KTFPB		o-NPOE	+	+
AS-D	TDMAC		DOS	+	−
AS-N	TDMAC		o-NPOE	+	+
PS-D	TBHDPB		DOS	+	−
PS-N	TBHDPB		o-NPOE	+	+
CARB-B	Carbonate ionophore VII	TDMAC	BOA	+	+
CARB-N	Carbonate ionophore VII	TDMAC	o-NPOE	+	+

KTFPB—potassium tetrakis [3,5-bis-(trifluoromethyl) phenyl] borate; TDMAC—tridodecylmethylammonium chloride; TBHDPB—tributylhexadecylphosphonium bromide; DOS—bis(2-ethylhexyl) sebacate; o-NPOE—2-nitrophenyl octyl ether; BOA—bis(2-ethylhexyl) adipate.
